# Polymeric Coatings with Electrolyzed Acidic Water: A Novel Approach to Extending Egg Shelf Life and Quality

**DOI:** 10.3390/polym17010084

**Published:** 2024-12-31

**Authors:** Gina Parra A, Claudia Clavijo, Alejandro Castillo, Rodrigo Ortega-Toro

**Affiliations:** 1 Food Science and Technology Program, Architecture and Engineering Faculty, Universidad de Pamplona, Pamplona 543050, Colombia; 2 Microbiology and Biotechnology Research Group, Basic Science Faculty, Universidad de Pamplona, Pamplona 543050, Colombia; clauclavijo@unipamplona.edu.co; 3 Texas A&M AgriLife Research Group, Food Science and Technology Department, Texas A&M University, College Station, TX 77843, USA; a-castillo@tamu.edu; 4 Food Packaging and Shelf Life Research Group, Food Engineering Department, Universidad de Cartagena, Cartagena 130015, Colombia

**Keywords:** electrolyzed water, coating, eggs, shelf life

## Abstract

Electrolyzed acidic water (EAW) contains hypochlorous acid as its active compound, which is a potent antimicrobial. It was encapsulated in polymeric coatings and applied to the surface of eggs. The antimicrobial activity and the ability to extend the shelf life of eggs at ambient temperature for 45 days were evaluated, by physical, microbiological, and sensory analyses. The analysis also included the evaluation of mechanical, thermal, and crystallinity properties and the interaction between the coating components and the eggshell. The results showed that eggs from young, middle-aged, and adult hens, encapsulated and coated with EAW, hydroxypropyl methylcellulose, polyvinyl alcohol, and chitosan, gained resistance and a glossy appearance. The thickness of the coating was 2.9 µm for young and adult hens’ eggs and 2.60 µm for those of old hens, as observed by SEM. Shelf life was extended to 45 days under refrigeration and more than 30 days at ambient temperature. Coated eggs were acceptable for 85% of the panelists compared to 57% acceptance of non-coated eggs. The encapsulation and coating with EAW as an antimicrobial agent improved the surface protection of commercial eggs, reduced albumen liquefaction, and maintained quality by acting as a barrier against air, thereby preserving sensory characteristics.

## 1. Introduction

The egg is one of the most complex products in terms of regulations and requirements for its export. The Food and Agriculture Organization of the United Nations (FAO) mentions that global egg production has doubled since the 1960s, making these foods highly sought after and among the most consumed in the world due to their nutritional quality and low fat content [1]. On the other hand, a large number of eggs is lost due to spoilage or waste. In Spain, it was reported that approximately 144 million eggs are lost each year due to consumer confusion regarding the expiration dates of batches [2].

Although the production of eggs of optimal quality starts from the early stages of producing adequate poultry farming animal welfare [3,4], the development of polymeric coatings based on proteins, lipids, and polysaccharides has further facilitated the maintenance of egg quality during storage [5]. In previous research [6], hydroxypropyl methylcellulose and citric acid were employed to demonstrate the influence on thermoplastic starch films, achieving excellent results for polymeric coatings. While coating with edible films creates thin layers to protect the surfaces of the egg, improving their properties, the inclusion of encapsulated active compounds adds further benefits, such as microbial inhibition and an overall extension in shelf life [5]. The use of antimicrobial coatings does not replace adequate refrigeration, which is a fundamental practice to preserve the quality and prolong the shelf life of foods. Some studies report the extension of egg shelf life under refrigerated storage by 30–40 days or a greater reduction in *Salmonella* Enteritidis compared to non-coated eggs [7,8]. Since, in some countries, it is not mandatory to keep them refrigerated, and they do not have these measures in place, ref. [7] also reported an extension in shelf life during non-refrigerated storage.

Additionally, the combination of antimicrobial treatments with EAW as an antimicrobial [9,10] and chitosan coatings has shown good results in improving egg quality during six weeks of storage [11]. Hydrophilic compounds, such as hydroxypropyl methylcellulose and polyvinyl alcohol, soluble in water and salts [12,13,14], make this research an innovative project aimed at contributing to society. Edible films or coatings made from biodegradable compounds based on proteins, lipids, or polysaccharides are also used and have been found to extend shelf life [5]. The edible coating can subsequently be consumed, benefiting the environment by reducing waste [15].

Similarly, these coating materials will help in food by serving as a barrier to moisture [16] and oxygen, associated with mass loss in food [17]. According to [13], hydroxypropyl methylcellulose (HPMC), with the formula C_3_H_7_O, is a methylcellulose that has been modified by treatments with alkali and propylene oxide. It is a hydrophilic, semi-synthetic, viscoelastic, non-ionic polymer derived from alkyl cellulose through a reaction with a mixture of methylene chloride and propylene oxide and a pH between 5 and 8 [15]. Polyvinyl alcohol (PVA), with the formula (CH_2_CH)_11_, is a synthetic, colorless, and odorless polymer that is soluble in water, prepared by the alcoholysis (hydrolysis and saponification) of polyvinyl acetate to eliminate acetate group. Chitosan, with the formula C_56_H_103_N_9_O_39_, is a biopolymer of amino polysaccharides composed of β-D-glucosamine and N-acetyl-D-glucosamine, with 99% degree of deacetylation and antimicrobial activity [17]. It is a white powder soluble in acid with a pH between 4 and 6 and is extracted through the partial deacetylation of chitin from the exoskeleton of crustaceans [18].

HPMC with gelatin has been used to improve the preservation of lulo [19], while [20] enhanced permeability, tensile strength, and elongation at break by using modified HPMC, PVA, and chitosan with bamboo fibers for bioactive packaging. Chitosan is used in the food industry as a thickener, emulsifier, and preservative, as well as in biomedicine, pharmacosmetics, and agriculture [21].

The objectives of this study were to develop an edible coating containing encapsulated hypochlorous acid and test this encapsulated coating (EC) for enhancing the quality and extending the shelf life of shell eggs.

## 2. Materials and Methods

### 2.1. Procurement of Eggs

The egg samples were collected directly from a FENAVI-approved farm that produced eggs from Isa Brown hens. The eggs were separated into 3 categories based on the age of their laying hens and labelled as young (22 to 30 weeks old), middle-aged (30–65 weeks old), or adult (70–90 weeks old). Each recollection time, the number of eggs corresponding to each different experiment was placed in their original cardboard packaging and were placed in hard chests to protect their integrity and then transported to the laboratory within 2 h of collection.

### 2.2. Obtaining and Encapsulating Hipochlorous Acid

Hypochlorous acid was obtained by producing EAW using the Zhengyi Technology Co Limited ROX-10WB (made in Shenzhen, China), water ionizer by running a potable water solution over the membrane and through the electrolysis of a NaCl solution at 260,000 mg/L, which was previously standardized. The EAW generation time in the water ionizer was adjusted to 20 min. Since the major component of this process was hypochlorous acid, followed by a small amount of hydrochloric acid [10], the verification of active chlorine compounds (expressed as HOCl) was achieved by determining active chlorine (mg/L) using a HACH 2800 spectrophotometer (made in New York, NY, USA), oxidation reduction potential (ORP) with a redox meter (PSE instruments, (Deutschland and distributed of Higielectronix LTDA), and pH with an OHAUS Starter 2100 pH meter (made in México City, México).

To encapsulate hypochlorous acid, the EAW was mixed in a coating of hydroxypropyl methylcellulose and polyvinyl alcohol, following the procedure described [22] with some modifications, including adding a second coating of chitosan at a low proportion.

Hydroxypropyl methylcellulose (HPMC, Eastchemlab, Shanghai, China) was added to EAW at a proportion of 1 g per 100 mL EAW. HPMC acts as a viscous agent that interacts well with salts, facilitating their interaction with electrolyzed water. Then, 1 g of polyvinyl alcohol (PVA, Merck-Schuchardt, Hohenbrunn, Germany, pH of 5–7 and a molecular weight of 72,000 g/mol [23,24]) was added to the EAW-HPMC emulsion to act as a thickener and emulsion stabilizer. Lastly, 1 g glycerol was added. The resulting emulsion was stirred in a hot plate with a magnetic stirrer (Lab-Line/Pyro-Magnestir, Melrose Park, IL, USA) for 10 min.

### 2.3. Preparation of the Coating

The preparation of chitosan solution followed the procedure described by [11] with some modifications, as follows: A 0.2% chitosan solution was prepared by adding 0.01 g of chitosan (Sigma-Aldrich, St. Louis, MO, USA, average molecular weight of 190,000) [25,26,27] to 5 mL of acetic acid, stirring in a hot plate with magnetic stirrer (Lab-Line/Pyro-Magnestir) at 45 °C for 10 min. Then, 1 mL of glycerol was added, and stirring continued for another 10 min. The solution was completed with electrolyzed water pH 3.3 up to 500 mL, stirred, and placed in a heat sterilizer with a timer at 50 °C for 45 min. After this time, it was cooled to 25 °C.

### 2.4. Application of Encapsulation and Coating

For this experiment, a total of 30 eggs of each hen’s age category (young, middle-aged, and adult) were obtained in 3 independent collection times, for a total of 90 eggs, and transported to the laboratory as described in Section 2.1.

The application of the encapsulation and coating was performed by the immersion method [3], submerging 30 eggs for 2 min in the encapsulating solution, then allowing them to dry for 10 min in a controlled environment in a biosafety cabinet. After drying, a second coating was applied for 2 min using the 0.2% chitosan solution, and it was left to dry. This second coating aimed to prevent moisture loss from the egg and the hydrophilic coating and contributed to the microbiological stability of the system. In this context, the capsule is understood as the system consisting of the eggshell cuticle, the hydrophilic coating, and the hydrophobic coating [11,22].

### 2.5. Analysis of the Encapsulation and Coating

#### 2.5.1. Color Analysis

To determine the color and brightness of the eggs, a colorimeter (chroma meter CR 400, Konica Minolta, sensing Americas, Inc., México City, México) was used on the 90 egg samples labeled 1, 2, and 3, respectively. The egg was brought close to the device, and the readings for coordinates a and b were extracted, representing the chromatic coordinates. These readings were then verified on the CIELAB color scale for greater accuracy. Additionally, the reading for L*, referring to lightness, was recorded, which pertains to the perception of distinguishing objects from white to gray and from light to dark.

Finally, to calculate the total color difference, the following formula was used [28]:

(1) $${\Delta E}_{\mathrm{ab}}^{*}=\surd {\left(L_{2}^{*}-L_{1}^{*}\right)}^{2}+{\left(a_{2}^{*}-a_{1}^{*}\right)}^{2}+{\left(b_{2}^{*}-b_{1}^{*}\right)}^{2}$$

where ΔE measures how the visual perception of differences between two colors changes, ranging from 0 (no perception by human eye) to 100 (complete difference in colors) [28].

#### 2.5.2. Mechanical Properties

The study of mechanical properties was conducted through uniaxial tensile testing and fracture behavior using a texture analyzer (Lloyd instruments, Ametek Inc., Bongor Regis, United Kingdom), with a three-point diameter setup (FG/TPB—Food 3-point bend jigs). The sample was placed on a 1.5 cm-wide bridge with support at two points, and force was applied at the center until the eggshell fractured. The testing conditions were a head speed of 1.5 mm/s and a force of 30 gf.

#### 2.5.3. Scanning Electron Microscopy (SEM) Examination

Through microscopic examination, the surface and cross-section of egg samples were examined using SEM, which provides high-resolution images of the sample surface. This technique is especially useful for analyzing the uniformity and quality of coatings and thin films in various industrial applications. For this purpose, a JEOL-JSM-7500F microscope (Tokyo, Japan) was used to analyze the encapsulated and coated egg samples that had been previously treated. The eggshell samples, approximately 1 cm^2^ in size, were placed on the specimen holder and observed under magnifications of 250×, 7000×, and 9000×, allowing for photographic documentation to visualize differences in the samples.

#### 2.5.4. Fourier Transform Infrared (FT-IR) Spectroscopy and X-Ray Diffraction

The analysis of changes in chemical conformation and potential interactions between the components of the systems was performed using Fourier transform infrared (FT-IR) spectroscopy. This technique is particularly useful for identifying functional groups and assessing molecular interactions, providing valuable information about the chemical structure of the compounds being analyzed. On the other hand, crystallinity patterns were determined using X-ray diffraction with a Bruker-D8 system. This technique allows for the characterization of the crystalline structure of materials, identifying crystalline phases and evaluating the purity and structural order of the compounds. X-ray diffraction is essential in the study of materials in various fields, including chemistry and materials science.

#### 2.5.5. Thermodynamic and Thermogravimetric Analysis

The state of thermodynamic change of the materials was evaluated using differential scanning calorimetry (DSC). The equipment used was a DSC 250 TA Instruments (Bengaluru, India), with a heating ramp of 10 °C/min up to 120 °C, followed by an isotherm at the same temperature for 5 min. The sample was then cooled at a controlled rate to −90 °C at 10 °C/min, maintaining the temperature for 5 min. Finally, the sample was reheated at 10 °C/min from −90 °C to 250 °C [29].

Thermogravimetric analysis (TGA) was carried out using a TGA 5500 TA Instruments (Bengaluru, India), applying a heating rate of 10 °C/min from ambient temperature up to 800 °C. During the process, a nitrogen atmosphere was maintained until 600 °C, at which point it was switched to oxygen. The TGA technique is used to evaluate the thermal stability and composition of materials by measuring changes in the mass of a sample as a function of temperature or time. This information is crucial for understanding the thermal properties of materials, as well as their behavior under heating conditions.

### 2.6. Determination of Quality and Safety

After applying the encapsulation and coating solution, an analysis of the eggs’ shelf life was conducted. For this, 16 trays of eggs were used, each containing 30 eggs. Eight trays were designated as control group (eggs without EC), while the other eight trays received the encapsulation and coating solution, referred to as group (eggs with EC). The eggs in groups without EC and with EC were stored at room temperature in a laboratory in Bucaramanga, Colombia (~28 °C, and 67% relative humidity) over a total period of 45 days, at intervals of 0, 2, 8, 15, 23, 30, 38, and 45 days; samples were collected and subjected to physical analyses, as described below. At the end of the storage (45 days), the eggs were subjected to microbiological analysis.

#### 2.6.1. Microbiological Analysis

Microbiological analyses included testing for food safety indicators (*Salmonella* [30,31] and *Listeria* spp.) and food quality indicators (mesophilic aerobes, Enterobacteriacea, and molds and yeasts) [32]. *Salmonella* analysis was conducted according to [33] ISO 6579:2017; whereas, testing *Listeria* spp. involved adding 25 g in 225 mL of *Listeria* enrichment broth (Merck, Darmstadt, Germany), incubating at 30 ± 2 °C for 24–48 h, and then, plating on PALCAM agar (Scharlau, Barcelona, Spain). For the enumeration of mesophilic aerobic microorganisms and molds and yeasts (shelf life), each sample consisted of one egg and was sampled by placing it in a bag with 90 mL of peptone water and mixed by sonication in an Emerson Branson 2800 sonicator (St. Louis, MO, USA) at 110 kHz for 10 min to increase microbial detachment. This suspension and two consecutive dilutions were then plated on the corresponding medium. For mesophilic aerobes, the samples were plated on plate count agar (Merck) and incubated at 37 ± 2 °C for 24–48 h. For molds and yeasts, the samples were plated on rose bengal agar (Roseto Degli Abruzzi, Italy) and incubated at 24 ± 2 °C for 5 d, and the enumeration of Enterobacteriaceae was performed by dilution plating on violet red bile glucose (VRBG) agar (Oxoid, Thermo Fisher, Waltham, MA, USA), incubating at 37 ± 2 °C for 24–48 h. Colony counts were performed and reported as CFU/g. These analyses were carried out in triplicate for each specified storage time.

#### 2.6.2. Physical Analysis

Physical analyses of the 32 eggs were conducted externally, assessing the shell for appearance, odor, texture, and weight using a PRECISA XB30OM Gravimetrics AG scale (Zurich, Switzerland). For size measurement, a stainless steel Spurtar Vernier caliper (Manufactured by King company of France), was used. For the internal assessment, the eggs were cracked open and placed in a Petri dish to measure the height and diameter of the yolk with a modified plastic caliper. The yolk index was calculated using the formula IY = AY/DY × 100, where IY = yolk index (mm); AY = yolk height (mm); DY: yolk diameter (mm) [34], and the Haugh unit was calculated with the formula HU = 100 × log (H − 1.7W^0.37^ + 7.6), where HU is the Haugh unit, H is the height of the albumen, and W is the weight of the egg. The resulting number determines the Haugh unit score as follows: >90 excellent, >80 good, >70 acceptable, and >65 fair [35].

Yolk color was measured using the Roche color fan, and the pH of the yolk and albumen was measured with a digital pH meter (OHAUS Starter 2100). This was carried out in triplicate, according to the previously indicated storage times, verifying the required parameters (Table 1).

As an element for comparison, the microbiological and physical characteristics of the egg were compared to criteria listed in Colombian standard NTC 1240:2011 [30], shown in Table 1 for shell eggs. However, except for *Salmonella*, this standard does not include all other microbial groups tested. For these parameters, criteria were selected from other sources [30,31] and are shown in Table 2. These criteria are solely a form of verification of the egg quality and have no relation with compliance with any regulation or lack thereof.

#### 2.6.3. Sensory Analysis

For the sensory characteristics, 160 eggs were used, evaluated by 20 semitrained judges who tasted boiled eggs for 10 min. The eggs were peeled and marked with codes as blind samples, with one being an uncoated egg (A) and the other a coated egg (B). They were stored for 8, 20, 30, and 45 days at room temperature (28 °C) and 67% humidity, using a survey form to rate the attributes, such as color, odor, flavor, and texture on a seven-point hedonic scale. Preference questions were included, based on the descriptors outlined by [36] to determine the degree of acceptability: strongly dislike = 1 (14%), moderately dislike = 2 (28%), slightly dislike = 3 (43%), neutral = 4 (57%), slightly like = 5 (71%), moderately like = 6 (85%), and strongly like = 7 (100%) [37].

#### 2.6.4. Statistical Analysis

The experimental design was a completely randomized block design with three repetitions and independent schemes. All microbiological count data, including NMP, were converted to Log base 10; the sensory analysis data were not subjected to any conversion. All data were confirmed to be normally distributed by a Shapiro–Wilk test and then analyzed using ANOVA with JMP Statistical Discovery LLC. software (Version 17), USA, with a confidence level of 95% and significance (*p*) ≤ 0.05. When the ANOVA indicated that there were significant differences between parameters, means separation was conducted using Tukey’s test.

## 3. Results

### 3.1. Encapsulate and Coating

Acidic electrolyzed water (AEW) was used as a diluent (97% of the formulation). The final formulation contained HPMC (1%) and PVA (1%) as encapsulating polymers and 1% glycerol added as a plasticizer, resulting in a soft gel that facilitated application on the surface of the eggs through direct immersion.

This formulation for coating the eggs provided a polymeric gel with excellent adherence to the egg surface via direct immersion.

Considering that eggs are one of the most important and economical protein sources for consumers, they are also regarded as a potentially hazardous food, which supports the growth of bacterial pathogens, such as *Salmonella*, and, therefore, need to be subjected to temperature control [38]. In a review of publications from 2013 to 2020 [4], it was determined that temperature treatments must be applied to ensure food safety in eggs. The hypochlorous acid used as an antimicrobial in this study has strong bactericidal power, but it is unlikely to reach bacteria if lodged inside the egg. However, surface disinfection of eggs can be an effective control measure, since the egg surface can be a source of pathogen internalization or the cross-contamination of other eggs or food contact surfaces. The authors of [9] identified 768 articles published between 2000 and 2022 that mention the use of electrolyzed water in the food industry. In this case, the capsule is understood to be the system consisting of the egg cuticle, the hydrophilic coating, and the hydrophobic coating [11]. The authors of [22] also demonstrated that the use of AEW as a coating allowed for a homogeneous surface and good cohesion between the materials.

### 3.2. Application of Encapsulation and Coating

The encapsulation and coating of the eggs were performed through several tests with sterile water as a diluent for the polymers, but it was found that the use of electrolyzed water directly allows for encapsulation in the polymers, facilitating the application process on the eggs via direct immersion, previously marked as young, middle-aged, and adult, with 30 eggs each, according to the age of the laying hens. The eggs show the gloss imparted after applying the encapsulation and coating, which can provide a more appealing appearance to consumers, corroborated by [39,40], who applied oil as a coating, and concurred that the resulting sheen may provide an improvement to the appeal of the eggs to the consumers.

### 3.3. Analysis of the Encapsulation and Coating

The analysis of the encapsulation and coating on the shells of the eggs from Isa Brown laying hens was conducted in the following order:

The mean L*, a*, and b* values in coated and non-coated eggs by hen age are shown in Table 3. There were no significant changes (*p* ≥ 0.05) in egg color between coated vs. non-coated eggs. This indicates that the coating does not cause any changes in color, meaning that the coating does not have a negative impact on quality. However, the gloss, which is caused by light reflection and not related to the actual color of the eggs, was evident (not shown in figures) and may be an added factor making the eggs more attractive to the consumers.

The fracture behavior of the mechanical properties highlighted that the coating in imparted hardness to the eggshell of eggs laid by young and middle-aged hens but not on eggs laid by adult (>70 weeks old). The ∆ hardness was as large as expected, but there was no effect of the hen age laying the eggs on the shell hardness when no coating was applied. In contrast, the shell was significantly harder on coated eggs that were obtained from young hens compared to adult hens. These data are shown in Table 4. The ∆ hardness (hardness in N on eggshell of control eggs—hardens on eggshell of coated eggs) was −2.5, −1.9 and 0.1 N for eggs from young, middle-aged and adult hens, indicating that the coating increased the hardness of the shell by 25 times eggs more in the young hen group than those of the adult hen group. This was also noted by [41], who related that, as hens age, they do not assimilate calcium, which is involved in the formation of the eggshell; thus, it is important to supplement these birds with an increased dietary intake of this element. Similarly, the authors of [40] confirmed the use of immersion coatings, employing mineral oil with excellent results in gloss, hardness, and quality.

**Figure 1:**
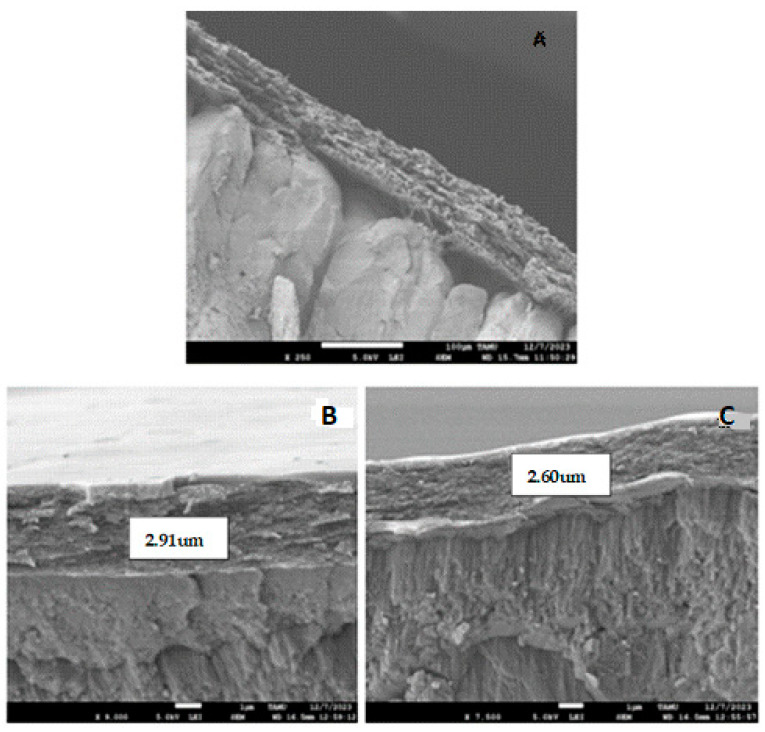
SEM images of cross-sectional views of eggshell surfaces after coating application on eggs laid by young, middle-aged, and adult hens. (**A**) View of an eggshell in the control sample, (**B**) view of an eggshell from an egg laid by middle-aged hens, and (**C**) view of an eggshell of an adult hen-laid egg.

The SEM images of the surface of the eggshell of eggs obtained from hens of varying ages are shown in Figure 1. Figure 1A shows the control sample with the typical fiber assemblies, covering the shell pores. Figure 1B,C clearly demonstrated that the coating did not penetrate the pores of the eggshell. In Figure 1B, the thickness of the coating on the eggshells in middle-aged hens was found to be 2.91 μm, providing resistance and protection against the high porosity of the shell. This same value was observed for young hen eggs (SEM image not shown). The coating largely covers the eggshell cuticle without pores or cracks (outer layer of the shell). These results are consistent with [22], where eggs observed in SEM maintained a smooth surface without pores. In contrast, the thickness of the coating on the eggshell of adult hen eggs (Figure 1C) was 2.60 μm, possibly due to a loss of smoothness in the egg, inhibiting the adhesion of the polymer layer. Further research should determine whether these differences affect the level of protection against microorganisms that these coatings provide. This is similar to the report by [42], who obtained bell-shaped microstructure images due to the calcium present in the eggshell, while commercial calcium appears in hexagonal form. The authors of [4] also presented the microstructure of the eggshell membrane in SEM, confirming the image obtained with the presence of fiber assemblies in the membrane. Likewise, the authors of [43] highlighted the eggshell membrane in SEM as a highly collagenized fibrous network, and the authors of [11] applied a chitosan coating, demonstrating that it also conferred thickness to the shell, with a protective barrier observed by SEM.

**Figure 2:**
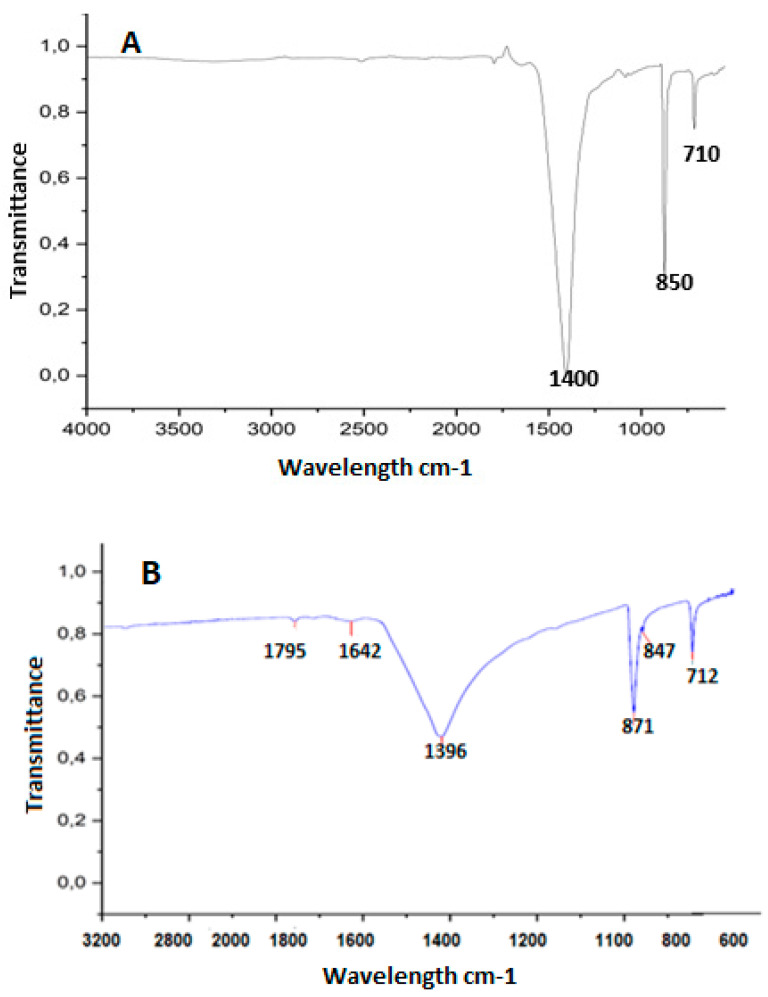
FTIR spectra on eggshell surfaces with encapsulation and coating. (**A**) spectra on control eggshells (no EC) and (**B**) spectra for eggshells with EC.

The study of changes in chemical conformation and possible interactions among the components of the systems was verified using Fourier transform infrared spectroscopy (FT-IR) (IR Prestige-21, Shimadzu, Tokyo, Japan), on the eggshell without EC and with EC on the encapsulation or coating (Figure 2).

On the control eggshells (Figure 2A), the highest peak observed was at 1400 cm^−1^, indicating the presence of calcium and carbon dioxide, followed by calcium carbonate, and in third place, calcium oxide [44]. As expected in the eggshell without coating, this spectrum was greater than on eggshells from eggs with EC (Figure 2B). In the eggshell with coating, salts and other components were observed in the spectrum in addition to calcium. These results confirm the findings of [41], who identified trace elements, such as magnesium in low concentration, phosphorus, sodium, and potassium. This was also highlighted by [45], who reported that the composition of the eggshell consisted of 96% calcium carbonate, 1% magnesium carbonate, and 1% calcium phosphate, along with organic materials and water. Figure 2B showed that the spectrum presented double bond peaks in the first and second spectra, corresponding to the wavelength range from 1795 cm^−1^ to 1680 cm^−1^, with the presence of anhydrides and alkenes. The third, fourth, fifth, and sixth peaks are in the fingerprint region of the vibrations, highlighting the presence of salts that could possibly provide resistance to the egg with coating. Other studies corroborate the presence of trace elements in the eggshell [41,43].

**Figure 3:**
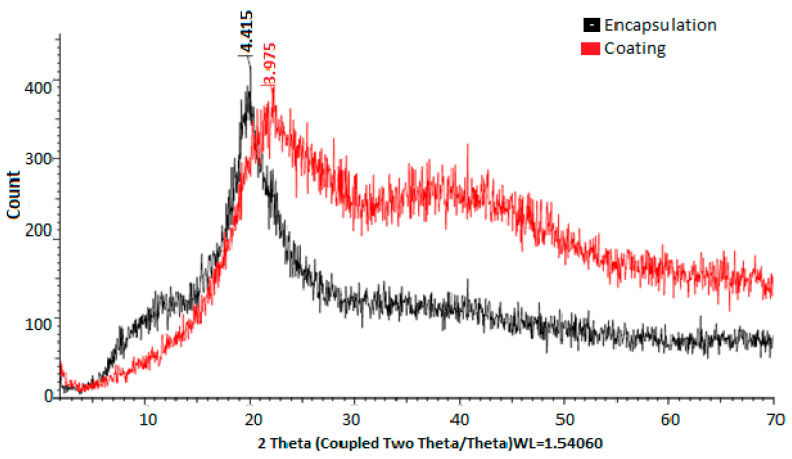
X-ray diffractograms of materials used for encapsulation and coating.

The crystallinity patterns were determined by X-ray diffraction, showing greater crystallinity of the material when chitosan was present in the coating (Figure 3).

**Figure 4:**
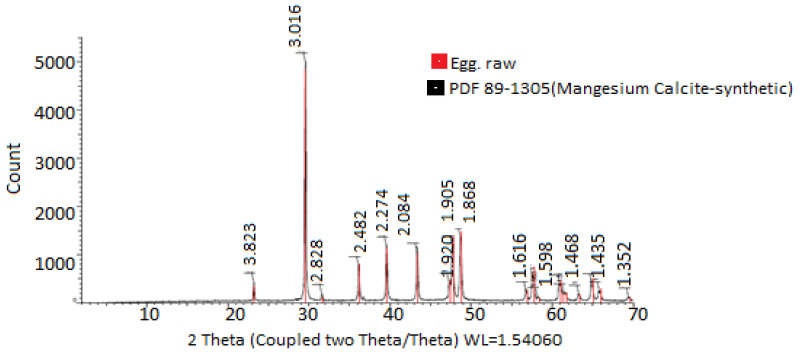
Crystallinity analysis of materials used for eggshell encapsulation and coating. The values were obtained from X-ray diffraction analysis.

In Figure 3, the X-ray diffractograms of the materials used for encapsulation were observed. The diffractogram of PVA combined with HPMC and electrolyzed water is shown in black, while the diffractogram of chitosan and electrolyzed water is shown in red. In the first case, peaks are observed at 4.415° 2θ, while in the second case, peaks are observed at 3.875° 2θ. Chitosan, in turn, shows a greater prevalence of both amorphous and crystalline regions. These observations are consistent with those reported by [46], who mentioned the use of chitosan compounds, highlighting their crystallinity and rigidity. In both cases, it was evident that the PVA + HPMC combination results in semicrystalline and more amorphous polymers, making them ideal for coatings [47,48]. The X-ray diffractogram of the eggshell in Figure 4 allowed for the verification of the crystallinity of the eggshell materials with coating. Peaks were observed at 3.823, 3.016, 2.828, 2.482, 2.274, 2.084, 1.920, 1.905, 1.868, 1.619, 1.598, 1.518, 1.468, 1.435, and 1.352° 2θ. The more peaks this figure has, the sharper they appear. A crystalline structure was also demonstrated in the eggshell with the coating, with the highest peak at 3.016° 2θ, corresponding to calcium. This is corroborated by [41,44,49,50].

**Figure 5:**
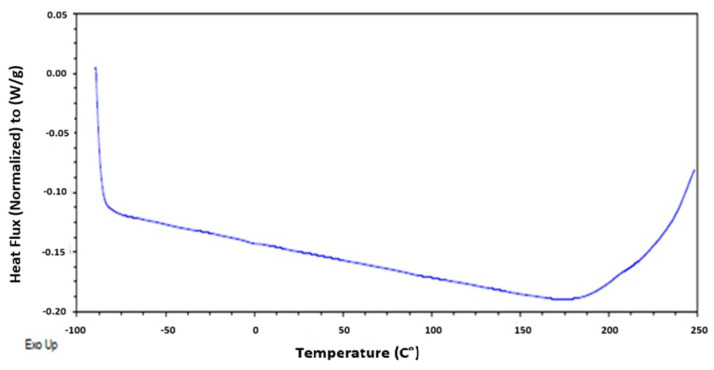
Heat flux of eggshell with EC, as determined by differential scanning calorimetry.

The thermodynamic state changes were evaluated using differential scanning calorimetry (DSC). In Figure 5, no glass transition, crystallization, or melting was observed. Additionally, no endothermic or exothermic processes were detected. However, a baseline shift was evident, which can be correlated with the possible thermal degradation of the material at 250 °C. This was corroborated by [18], who observed the degradation of eggshells in several steps, with two stages of weight loss occurring at temperatures between 25 °C and 900 °C.

**Figure 6:**
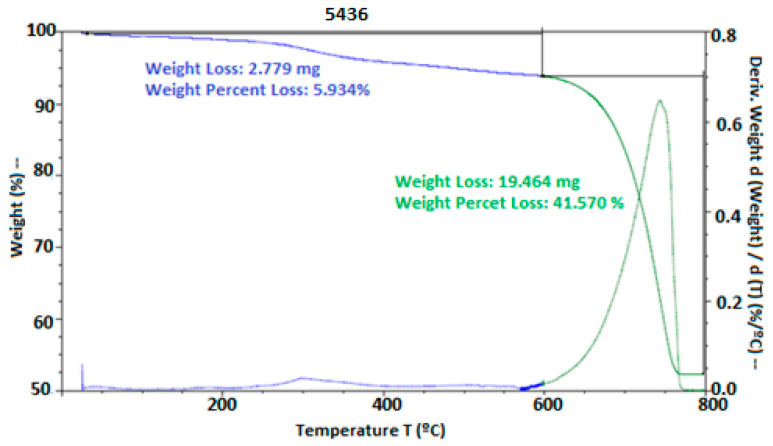
Thermal stability of the eggshells with EC. Thermal stability was expressed by weight loss using thermogravimetric analysis.

Thermal stability was evaluated through thermogravimetric analysis (TGA). In Figure 6, thermal degradation was observed starting at 250 °C and 300 °C for the materials HPMC, PVA, and chitosan, while, in the presence of hypochlorous acid, degradation began at 30 °C. However, at 780 °C, the thermal degradation of the eggshell occurred, where the calcium salts in the material degraded at this temperature. According to [41], these temperatures were observed during the degradation of the eggshell. They also mentioned that the peak obtained in the TGA of the eggshell appeared between 510 and 745 °C, possibly due to the transformation of Ca into CaCO_3_, releasing CO_2_. Other studies on thermal degradation, such as those by [4], who used HPMC as coating films, demonstrated that these can be used without complications, and [17], who used a chitosan, eugenol, and oregano coating on cheese, showed the formation of soluble films with antimicrobial properties. Similarly, ref. [49] highlighted that differential scanning calorimetry (DSC) with an acid coating allowed for the incorporation of a high-moisture matrix, presenting two endothermic peaks, one exothermic peak, and a glass transition, with microbial inhibition.

### 3.4. Determination of Quality and Safety

#### 3.4.1. Microbiological Analysis

**Figure 7:**
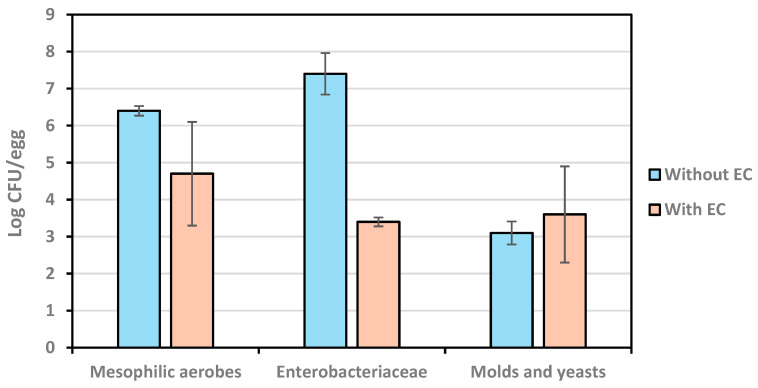
Microbial counts on eggs with and without coating at the end of a 45 d storage.

The results described in Figure 7 show a significant (*p* < 0.05) effect of the application of AEW as an encapsulant and coating against mesophilic aerobes and Enterobacteriaceae. At the end of storage for 45 d at room temperature, the counts of mesophilic aerobes and Enterobacteriaceae were, respectively, 1.7 and 4.0 log cycles lower on eggs that were treated with the AEW-based coating vs. non-treated controls. In contrast, there was no statistical difference (*p* ≥ 0.05) in the counts for molds and yeasts between coted and non-treated eggs (3.1 and 3.6 log CFU/g controls and coated eggs, respectively), indicating that the antimicrobial in the coating did not show an inhibitory effect against these organisms during storage. The reasons for this difference remain to be explained and will be the focus of future research.

When these counts were compared to example criteria obtained from the literature, the mean counts of all three quality indicators were above their recommended maximum values (see Table 2). However, this comparison must be used cautiously, since these reference values were for heat-processed egg and are not applicable to fresh shell eggs for the purposes of making decisions about the acceptability of the coated eggs [35]. Still, the protection of eggs against microbial growth was demonstrated. A factor to keep in consideration was that chitosan has antimicrobial activity as well. Therefore, from these data, the antimicrobial effect attributed exclusively to the hypochlorous acid contained in AEW is uncertain. Other authors have also combined AEW with chitosan in coatings with a similar level of protection [50]. Similarly, the authors of [11] used AEW as a disinfectant and chitosan as a coating on eggs, successfully preserving their quality for 42 days of storage. Despite this, the coating improved egg quality due to its antimicrobial effect, extending the shelf life of eggs to more than 30 days at room temperature. In the analysis of *Salmonella* spp. and *Listeria*, none of these organisms was detected in any sample, reporting the absence of *Salmonella*/25 g and the absence of *Listeria*/25 g. *Listeria monocytogenes* is not a pathogen of interest in fresh shell eggs, and the testing for *Listeria* spp. in this study was to examine the antimicrobial effect of the AEW-based coating against a variety of microorganisms. Our results are consistent with [51].

#### 3.4.2. Physical Analysis

Physical analyses demonstrate that eggs with encapsulation and coating maintain a higher quality stability than those without encapsulation or coating during storage for 45 days. The pH of both the yolk and albumen was also preserved in eggs with encapsulation and coating, as confirmed by [52], who showed that the use of coatings on eggs, particularly when applied to the entire egg, improves their internal quality (Table 5).

The results described in Table 5 showed that the use of coatings prevents contaminants from entering and maintains the stability of the egg albumen. This was also demonstrated by [53], who applied coatings, in this case 5% NaClO, maintaining albumen stability after thermal treatment at 56 °C and storage for 30 days. In terms of weight, there was a lower weight loss in eggs with encapsulation and coating, both refrigerated and non-refrigerated, compared to the other eggs. Similarly, ref. [54] treated commercial eggs with a rice protein coating enriched with essential oils, storing them at 20 °C for 6 weeks, reducing weight loss and improving the internal quality of the eggs compared to eggs without coatings. However, quality diminishes in room temperature [55] over time during storage, as normal chemical processes generate CO_2_, leading to weight loss in eggs with coatings [56] and retarding the deterioration in the egg yolk [57].

The results for the yolk index and Haugh unit show that the internal quality is preserved in refrigerated eggs with coating for up to 45 days of storage. In contrast, eggs with coating at room temperature (25 °C) maintained acceptable quality for 38 days, while eggs without coating reached acceptable quality at 30 days, according to this study [57]. The yolk index shows lower values with longer storage time, as the vitelline membrane fiber loses resistance, which is more pronounced in eggs without coating. Additionally, the color measured using the Roche scale remained more stable in eggs with encapsulation and coating compared to the others. Likewise, Haugh unit values of >70 indicated acceptable quality, according to the quality table [35]. This was corroborated by [58], who investigated egg quality using the Haugh unit over fifty weeks of laying.

Regarding the eggshell, all samples showed a characteristic egg smell, slightly stronger in the without EC samples. As for the texture of the eggshell, the samples with EC were smoother than the without EC samples.

#### 3.4.3. Sensory Analysis

The sensory analysis was performed with 20 semitrained panelists using blind triangular coded samples, where one sample was without coating and encapsulation (EC), and another sample was with coating and encapsulation (EC), stored for 8, 20, 30, and 45 days, considering the descriptors I strongly dislike = 1, I moderately dislike = 2, I slightly dislike = 3, neither like nor dislike = 4, I slightly like = 5, I moderately like = 6, and I strongly like = 7. The results, including the means and standard deviations, are shown in Table 6.

It was demonstrated that there was no significant difference (*p* > 0.05) in smell and taste between samples without EC and with EC. However, the texture and color of the sample without EC were more accepted by the judges than the sample with EC. The results allowed for the differentiation that, after 20 days of storage at ambient temperature (25 °C), there were no changes in taste between the sample without coating and the sample with coating (*p* > 0.05), where no significant difference in smell and taste was noted. Nevertheless, in terms of texture, color, and smell, the sample with EC was preferred by the judges.

On day 30, a preference was observed for the sample without EC regarding texture. However, in the sample with EC, the other attributes of color and taste received higher scores, possibly because the coating gave the egg a fresher feel. Similarly, for the smell attribute, both samples demonstrated no significant difference (*p* > 0.05).

On day 45, the preference for color, taste, and texture was more pronounced in the sample with EC, possibly because the coating imparted greater freshness to the egg. In contrast, the sample without EC retained a stronger egg smell, which was less intense in the sample with EC, likely due to the coating. Acording to [56], taste plays a pivotal role in determining egg quality. However, with EC, eggs were acceptable for 85% of the panelists compared to the 57% acceptance of eggs without EC.

## 4. Discussion

The results of this study provide valuable insights into the potential of starch-based films as effective coatings for preserving the quality and extending the shells of the eggs.

The polymeric encapsulates using HPMC and PVA were developed for the encapsulation of AEA. When applied as a chitosan polymeric coating on eggs, these encapsulates showed a notable reduction in *Salmonella* spp. This effect was more significant at refrigeration temperatures compared to ambient temperature; although, no significant differences were observed between both temperatures. Additionally, the coating provided extra protection and strength to the egg’s surface, improving its quality properties and inhibiting microorganism growth. This allowed for an extended shelf life of over 30 to 36 days at room temperature (26–28 °C) and more than 40 to 45 days under refrigeration (4–6 °C). Over time, this protection also helped reduce albumen liquefaction by acting as a barrier to air, preserving the egg’s sensory characteristics, such as color, taste, and texture, though a preference for the smell of eggs without coating was noted, as they maintained their characteristic aroma. These findings suggest that the use of encapsulated and polymeric coatings based on electrolyzed water, HPMC, PVA, and chitosan not only improves egg quality but is also supported by analyses of mechanical, thermal, crystallinity, and component interactions presented in this study.

## 5. Conclusions

In conclusion, this study shows that the use of electrolyzed water obtained through electrolysis, when encapsulated and applied as a polymeric coating, proves to be an effective strategy for enhancing egg safety and quality, indicating that this coating significantly reduces *Salmonella* spp., offering protection to the eggshell and improving its physical and sensory properties, inhibiting microorganisms, and extending its shelf life. These findings underscore the potential of hypochlorous acid as an innovative solution for food preservation.

## Figures and Tables

**Table 1:** Standards for shell eggs NTC 1240:2011.

Microbiological Requirements			
Parameter	Result	Method	Reference
*Salmonella* spp.	Negative in 25 g of sample	International Standard ISO 6579:2017 [33]	NTC 1240 2nd update [30]
Physical Characteristics			
Parameter	Specifications		
Appearance	Oval shape, clean shell (according to NTC 1240), without deformations		
Color	Characteristic of the genetic line, white interior and orange yolk (Roche color fan)		
Odor	Characteristic of the product		
Texture	Smooth shell, semisolid interior		
Freshness	Yolk index (measured on a flat surface, height by diameter)		
Shell	Whole (without visible cracks or fissures). Clean. If there is dust, bird droppings, or egg remnants, this must be less than or equal to 25% of the shell surface Color characteristic depending on the genetic line of the bird.		

**Table 2:** Microbiological criteria.

Parameter	n	c	m	M
Mesophilic aerobes	5	2	10^4^ CFU/g or mL	5 × 10^4^ CFU/g or mL
Enterobacteriaceae	5	2	10 CFU/g	10^2^ CFU/g
*Salmonella* spp.	5	0	Absence/25 g or mL	-----
*Listeria monocytogenes*	5	0	Absence/25 g or mL	-----
Molds and yeasts	5	2	10 CFU/g	10^2^ CFU/g

Note: [30,31,32] for *Salmonella* and *L. monocytogenes*. n: Number of units in the sample to be analyzed. c: Maximum number of sample units that can contain a number of microorganisms between m and M for the food to be of acceptable quality. m: Maximum permissible microbiological limit to identify a good quality level. M: Concentration that separates the acceptable quality level from unacceptable safety.

**Table 3:** Effect of the EC on the color values of egg laid by hens of different ages.

		Mean Values of Egg Laid by					
Samples		Young Hens		Middle-Aged Hens		Adult Hens	
		Before EC	After EC	Before EC	After EC	Before EC	After EC
Color	L*	59.06 ± 3.4A *^a^*	59.40 ± 3.3A	58.10 ± 2.8Aa	56.77 ± 2.8Aa	55.74 ± 4.4Aa	55.87 ± 4.2Aa
	a*	21.45 ± 2.1A	21.14 ± 1.9A	22.23 ± 1.4A	22.47 ± 2.4	23.45 ± 2.8A	23.60 ± 1.8A
	b*	29.96 ± 1.3A	29.57 ± 1.4A	30.09 ± 0.5A	29.89 ± 1.0	31.39 ± 0.3	31.32 ± 0.3
	∆E*	69.61	69.64	69.10	69.66	68.13	68.26

*^a^* There were no effects of the coating application or age of hens in color attributes.

**Table 4:** Egg firmness in egg with and without EC.

	Hardness (Mean ± STDEV) in N		
Laying Hen’s Age Group	No EC ^a^	With EC	∆ ^b^
Young	30.5 ± 1.2Ba ^c^	32.9 ± 1.7Ab	−2.5
Middle-aged	29.4 ± 1.5Ba	31.3 ± 1.1Aab	−1.9
Adult	29.6 ± 1.5Aa	29.6 ± 2.3Aa	0.1

^a^ EC = encapsulation coating material, ^b^ ∆ = shell hardness (N) on non-coated eggs—shell hardness on coated eggs ^c^. Within rows, means followed by same capital letter are not significantly different. Within columns, means followed by same small letter are not significantly different. *p* ≥ 0.05.

**Table 5:** Physical analysis of eggs with and without coating.

Storage (Days)	Treatment	Storage Conditions	Yolk pH	Albumen pH	Yolk Color	Yolk Index	Haugh Unit	Weight (g)
0	Control	Ambient	6.5	9.9	12	43.2	82.7	61.1
	Control	Refrigeration	6.5	9.7	12	43.2	82.7	61.3
	E + C	Ambient	6.4	9.9	11	43.2	82.6	61.2
	E + C	Refrigeration	6.4	9.7	12	43.2	82.9	61.5
2	Without EC	Ambient	6.5	9.8	12	43.2	80.3	58.9
	Without EC	Refrigeration	6.5	9.6	12	43.2	82.7	61.3
	E + C	Ambient	6.4	9.8	11	43.2	80.6	59.2
	E + C	Refrigeration	6.4	9.5	12	43.2	82.9	61.5
8	Without EC	Ambient	6.6	9.8	12	43.2	80.7	59.3
	Without EC	Refrigeration	6.5	9	12	43.2	82.7	61.3
	E + C	Ambient	6.4	9.7	11	43.2	80.9	59.5
	E + C	Refrigeration	6.4	9.4	12	43.2	82.9	61.5
15	Without EC	Ambient	6.6	9.6	11	41.6	79.6	58.2
	Without EC	Refrigeration	6.5	8.6	12	42.5	82.7	61.3
	E + C	Ambient	6.4	9.6	12	43.2	79.8	58.4
	E + C	Refrigeration	6.5	9.3	12	43.2	82.9	61.5
23	Without EC	Ambient	6.7	9.3	11	40.5	71.7	60.4
	Without EC	Refrigeration	6.6	8.4	12	41.6	72.4	61.1
	E + C	Ambient	6.5	9.5	12	42.5	71.9	60.6
	E + C	Refrigeration	6.6	9.2	12	42.5	72.8	61.5
30	Without EC	Ambient	6.8	8.7	11	39.2	71.9	60.6
	Without EC	Refrigeration	6.6	8.3	12	40.5	72.3	61
	E + C	Ambient	6.5	9.4	12	42.5	78.8	60.8
	E + C	Refrigeration	6.6	9.1	12	42.5	82.8	61.4
38	Without EC	Ambient	6.8	8.5	11	36	69.3	58.7
	Without EC	Refrigeration	6.7	8.2	11	37.7	71.5	60.9
	E + C	Ambient	6.5	9.3	11	40.5	74.9	58.9
	E + C	Refrigeration	6.6	9	12	41.6	76.3	61.3
45	Without EC	Ambient	6.8	8	12	32	63.2	60.2
	Without EC	Refrigeration	6.7	8.2	11	34.1	68.8	60.8
	E + C	Ambient	6.6	9.2	11	40.6	73.4	60.4
	E + C	Refrigeration	6.6	9	12	40.4	75.1	61.1

**Table 6:** Sensory analysis of boiled eggs with and without coating.

Days of Storage								
	8		20		30		45	
Attributes	Without EC	With EC	Without EC	With EC	Without EC	With EC	Without EC	With EC
Color	6.4 ± 0.99 ^a^	5 ± 1.31 ^a^	5.65 ± 0.98 ^a^	5.7 ± 1.3 ^a^	4.5 ± 1.27 ^a^	5.9 ± 1.05 ^a^	5.4 ± 0.94 ^a^	6 ± 0.64 ^a^
Smell	5.5 ± 1.14 ^a^	5.5 ± 0.99 ^a^	4.95 ± 1.43 ^a^	5 ± 1.52 ^a^	5.3 ± 1.68 ^a^	5.3 ± 1.34 ^a^	4.9 ± 1.19 ^b^	4.6 ± 1.04 ^b^
Flavor	5.8 ± 1.3 ^a^	5.4 ± 1.1 ^a^	5.6 ± 1.27 ^a^	5.4 ± 1.75 ^a^	5.1 ± 1.31 ^a^	5.9 ± 0.94 ^a^	3.0 ± 1.2 ^b^	6 ± 0.58 ^b^
Texture	6 ± 1.2 ^b^	5.8 ± 1.2 ^b^	5.6 ± 1.14 ^b^	5.7 ± 1.52 ^b^	6.2 ± 0.89 ^b^	5.1 ± 1.1 ^b^	5.8 ± 1.4 ^b^	6.1 ± 0.78 ^b^

^a^ Within columns, means followed by same small a letter, are not significantly different. *p* ≥ 0.05, and ^b^ significantly different.

## Data Availability

The data are available upon request to the authors via e-mail.
